# A Novel Recombinant DNA System for High Efficiency Affinity Purification of Proteins in *Saccharomyces cerevisiae*

**DOI:** 10.1534/g3.115.025106

**Published:** 2015-12-29

**Authors:** Brian H. Carrick, Linxuan Hao, Philip J. Smaldino, David R. Engelke

**Affiliations:** * Department of Biological Chemistry, The University of Michigan, Ann Arbor, Michigan, 48109-0600

**Keywords:** CelTag, *Saccharomyces cerevisiae* protein purification, CBM3, myc epitope, affinity purification

## Abstract

Isolation of endogenous proteins from *Saccharomyces cerevisiae* has been facilitated by inserting encoding polypeptide affinity tags at the C-termini of chromosomal open reading frames (ORFs) using homologous recombination of DNA fragments. Tagged protein isolation is limited by a number of factors, including high cost of affinity resins for bulk isolation and low concentration of ligands on the resin surface, leading to low isolation efficiencies and trapping of contaminants. To address this, we have created a recombinant “CelTag” DNA construct from which PCR fragments can be created to easily tag C-termini of *S. cerevisiae* ORFs using selection for a *nat1* marker. The tag has a C-terminal cellulose binding module to be used in the first affinity step. Microgranular cellulose is very inexpensive and has an effectively continuous ligand on its surface, allowing rapid, highly efficient purification with minimal background. Cellulose-bound proteins are released by specific cleavage of an included site for TEV protease, giving nearly pure product. The tag can be lifted from the recombinant DNA construct either with or without a 13x myc epitope tag between the target ORF and the TEV protease site. Binding of CelTag protein fusions to cellulose is stable to high salt, nonionic detergents, and 1 M urea, allowing stringent washing conditions to remove loosely associated components, as needed, before specific elution. It is anticipated that this reagent could allow isolation of protein complexes from large quantities of yeast extract, including soluble, membrane-bound, or nucleic acid-associated assemblies.

Proteomics requires quick, specific, and dependable methods for purifying proteins and complexes. Typically, techniques for characterizing protein complexes require large quantities of highly purified protein, which is difficult to obtain for rare or unstable complexes. The most common method currently available for specific and pure protein purification in yeast is the dual affinity tag, known as TAP (tandem affinity purification) tag (Rigaut *et al.* 1999). This tag uses the IgG binding domain of protein A of *Staphylococcus aureus* (ProtA) (Uhlen *et al.* 1983; Nilsson *et al.* 1987) and a calmodulin-binding peptide separated by a Tobacco Etch Virus (TEV) protease cleavage site (Dougherty *et al.* 1989; Kapust *et al.* 2001). The tag is added on to the C-terminal end of proteins, which is bound to IgG immobilized on a bead matrix during the first step of purification. The bound protein is eluted from the matrix by TEV protease cleavage under mild conditions, and then is bound to calmodulin-coated beads in the presence of calcium during the second purification step. The bound proteins are then eluted from the calmodulin beads with EGTA (Lohman *et al.* 1989; Puig *et al.* 2001; Van Driessche *et al.* 2005).

Tandem affinity methods are very powerful, yet have limitations. Native protein complexes are often rare and unstable, and therefore without rapid, high yield purification methods these complexes might not be isolated cleanly using standard purification techniques. Often, *in vivo* overexpression of rare protein complexes is impractical because of artificial interactions and low concentrations of physiologically relevant partners. Such complexes, whether soluble, bound to specific genes, or associated with membranes, can exist as a few copies per cell, making isolation on a large scale mandatory. The problems with current approaches are that affinity purification reagents are expensive on a large scale, and the low concentration of affinity ligand per surface area/volume on affinity resins makes specific binding inefficient, leading to high signal-to-noise ratios. We sought to create a new recombinant DNA system for adding affinity tags to proteins that would address these issues.

The result is an affinity tag that provides specificity and extensive enrichment while also maintaining a relatively low cost, especially during the first, bulk isolation step. To do this, we created a C-terminal dual tag (termed CelTag) consisting of a family 3 cellulose binding module (CBM3) (Levy and Shoseyov 2002) and a 13x c-Myc repeat epitope tag (Terpe 2003) separated by a TEV protease cleavage site (Dougherty *et al.* 1989) (Figure 1). CBM3 was chosen due to its high affinity and specificity for a cellulose matrix and it has been shown to form an independent domain at either terminus of a protein (Stofko-Hahn *et al.* 1992; Levy and Shoseyov 2002; Hong *et al.* 2007). Cellulose is an excellent matrix for purification due to its very low cost, low background due to the few proteins that have nonspecific affinity for it (in yeast and animals), and its stability in a number of buffer conditions and pH levels. Recently, it was shown that CBM3 could be used as a single affinity tag to purify overexpressed proteins from recombinant DNA in *Pichia pastoris* (Wang and Hong 2014). A 13x c-myc repeat epitope was added in our constructs due to the availability and relatively low cost of strong monoclonal antibodies produced against this sequence. This could be particularly useful for lesser studied proteins in which only poor or no antibodies exist to study them.

**Figure 1:**
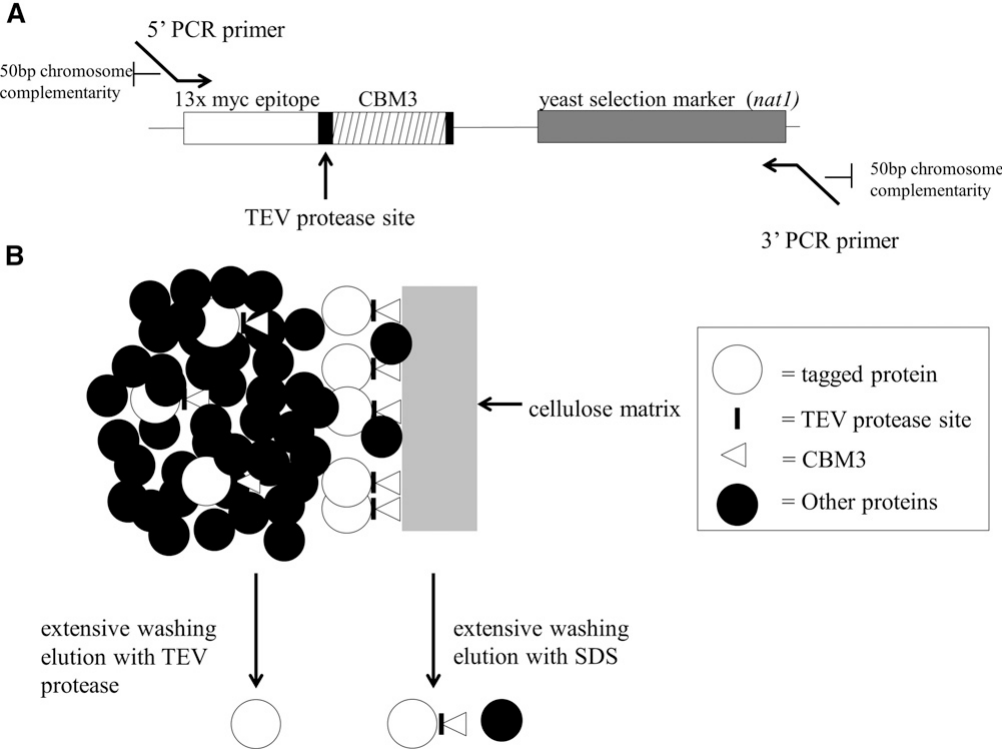
Purification scheme. (A) Line drawing of CelTag fragment from CelTag plasmid. PCR (polymerase chain reaction) of fragment was performed using primers with 50 nucleotides of sequence identity to the site of chromosomal insertion and 21 nucleotides of complementarity to the CelTag. The sequences of fragment and primers used in this study are found in File S1 and Table S1, respectively. (B) Schematic purification scheme. Pgk1 with C-terminal CelTag binds efficiently to cellulose surface and elutes specifically with TEV (Tobacco Etch Virus) protease. Elution with SDS (sodium dodecyl sulfate) results in low, variable levels of contamination from nonspecifically bound proteins. CBM3, family 3 cellulose binding module.

A major advantage of yeast for this type of study is that the transformed DNA sequences recombine into the yeast chromosomes by sequence homology (Figure 1) at a relatively high frequency. This makes it possible to easily tag the C-termini of proteins and analyze them *in vivo* in the proper physiological context. To determine the effectiveness and efficiency of integration, a marker is engineered into the tag, which allows for the yeast transformants to be isolated by growth on selective media. Here, we provide a proof of concept example of isolation of a soluble yeast protein.

## Materials and Methods

All reagents and chemicals are reagent grade and purchased from Sigma-Aldrich (St. Louis, MO) and Fisher Scientific (Pittsburgh, PA) unless otherwise noted.

### Strains

*Escherichia coli* DH5α strain (F– Φ80*lac*ZΔM15 Δ(*lac*ZYA-*arg*F) U169 *rec*A1 *end*A1 *hsd*R17 (rK–, mK+) *pho*A *sup*E44 λ– *thi*-1 *gyr*A96 *rel*A1) was used as the host cell for plasmid manipulation. Luria-Bertani (LB) medium with or without 100 µg/mL ampicillin was used to culture *E. coli* host cells or transformants. *Saccharomyces cerevisiae* BY4741 (MATα *his*3∆1, *leu*2Δ, *met*15∆, *ura*3∆) was used as a parent strain for transformation with DNA fragments as well as for recombinant protein expression. YPD medium (1% yeast extract, 2% peptone, and 2% dextrose) with or without 150 µg/mL of clonNAT (Werner BioAgents, Meisenweg, Jena, Germany) was used to culture *S. cerevisiae* parent strains and transformants.

### Plasmid preparation

All plasmids constructed in this study were isolated from DH5α cells through QIAGEN mini or midi prep kits according to procedures outlined in the QIAGEN Plasmid Purification Handbook (https://www.qiagen.com/resources/download.aspx?id=46205595-0440-459e-9d93-50eb02e5707e&lang=en).

### Construction of CelTag plasmid

A PCR fragment containing the 13x myc epitope repeat and clonNAT resistance gene was created from the digestion of the pFA6a-13myc-natMX6 plasmid (gift from Dr. Antony Carr at the University of Sussex) (Van Driessche *et al.* 2005) with restriction enzymes *Not*1 and *Cla*1 (New England Biolabs, Ipswich, MA) at 37° overnight. The fragment was then ligated into the pRS426 plasmid (Sikorski and Hieter 1989) which was cut with the same enzymes. A fragment containing the CBM3 was obtained from the pCIG plasmid (gift from Dr. Jiong Hong at Virginia Polytechnic Institute and State University) (Hong *et al.* 2008) and gap repaired between the 13x myc repeat and clonNAT resistance gene (see Supporting Information, File S1 for complete sequence). To avoid carrying over a yeast-compatible expression plasmid when transforming yeast with amplified DNA fragments, the tag was moved from pRS426 (shuttle vector for yeast and bacteria) to the pGEM-T Easy vector (Promega, Madison, WI) (a bacterial only plasmid). A DNA fragment containing the entire tag and selection marker was PCR-amplified from the pRS426 vector by PCR and was ligated into the pGEM-T Easy vector according to manufacturer’s specifications (https://www.promega.com/resources/protocols/technical-manuals/0/pgem-t-and-pgem-t-easy-vector-systems-protocol/). Correct sequence of the tag was confirmed by Sanger sequencing (University of Michigan Sequencing Core). The plasmid will be available through Addgene (Plasmid #66562) and the full plasmid map is provided in Figure S1.

### DNA fragment purification

Fragments of interest for construction of the CelTag plasmid and for the creation of tagged ORFs were isolated by running approximately 25 µg of DNA on a 0.8% agarose electrophoresis gel containing 20 mg/mL of ethidium bromide for visualization of the DNA. Gel bands of interest were quickly excised under long wave UV-light and DNA was electroeluted from the gel piece by sealing it in Spectra/Por Dialysis Membrane (MWCO 12000-1400) (Spectrum Labs, Rancho Dominguez, CA) and placed in 0.5× TBE buffer at 150 V until no ethidium bromide was visible in gel slices. The DNA was then extracted using phenol/chloroform and precipitated with NaOAc and EtOH.

### PCR of CelTag fragment

Primers were designed as follows. The forward primer contains 50 bp of homology to the end of the ORF where the fragment is to be inserted (excluding the stop codon), followed by 25 bp of complementarity to the beginning of the CelTag recombinant DNA construct (Figure 1). The reverse primer is 50 bp of homology to sequences downstream of the target ORF (∼25 nucleotides downstream from the stop codon), with 25 nucleotides complementary to the end of the CelTag fragment (see Figure S2 and Table S1 for sequences). The tag was amplified from the plasmid with the homologous ends to the ORF of interest and was purified by electrophoretic DNA purification and extraction as described above. To ensure there was enough fragment for efficient transformation into yeast, a total of 400 µL of PCR was done (4 × 100 µL reactions).

### PCR conditions

Optimum PCR conditions vary slightly with different primers, but were approximately as follows. Reaction consisted of Pfu Buffer (1X: 10 mM KCl, 6 mM (NH_4_)_2_SO_4_, 2 mM MgCl_2_, 20 mM Tris-HCl pH 8.8, 0.1% Triton X-100, and 0.1 mg/mL BSA), dNTPs (100 µM each), primers (1 µM each), Taq polymerase (Engelke *et al.* 1990) (5 units), Pfu DNA polymerase (Lu and Erickson 1997) (1 unit). Cycles: (94°, 5 min) × 1 cycle; (94°, 30 sec), (48°, 30 sec), (72°, 3 min) × 5 cycles; (94°, 30 sec), (53°, 30 sec), (72°, 3 min) × 30 cycles; (72°, 7 min), (4°, hold).

### Transformations

Saturated 5 mL overnight cultures of BY4741 grown in YPD were used to seed 22 mL cultures of YPD to an OD_600nm_ of approximately 0.2. Cultures were grown at 30° until the OD_600nm_ was 0.6–0.8 (midlog phase). Ten mL of cells were aliquoted for each transformation and were spun at 3500 rpm (Sorvall T 6000 B) for 5 min and washed twice with 100 mM lithium acetate (LiOAc). Pellets were then resuspended in 100 µL of 100 mM LiOAc. Ten µg of salmon sperm DNA and approximately 25 µg of purified PCR fragment containing the CelTag fragment with ends homologous to chromosomal sequences were added to the transformation reaction and incubated at 30° for 15 min. Next, 600 µL of PEG Mix (100 mM LiOAc, 50% PEG 3350) was added and mixed well before incubation at 30° for 30 min. Then, 68 µL of DMSO was added and the reactions heat shocked at 42° for 15 min. Cells were spun at 3500 rpm for 5 min and resuspended in 1 mL of YPD media and then grown at 30° for 4 hr. The cells were then spun at 3500 rpm for 5 min and resuspended in 250 µL of dH_2_O before plating to YPD + 1.5× clonNAT (150 µg/mL clonNAT). Positive clones were screened for by PCR over insertion junctions as well as visualization of myc-epitope tagged protein by western blot analysis using an anti-Myc-repeat antibody (9E10) (Santa Cruz Biotech).

### Yeast cell lysate preparation

Control or transformed yeast cells in 6 L of YPD medium were grown to early log phase, an OD_600nm_ of approximately 0.2 (either wild-type BY4741 or with tag inserted and therefore clonNAT resistant). Cells were harvested at 4000 rpm for 5 min at 4° (Sorvall RC 5C Plus centrifuge using SLA-3000 rotor), resuspended in 300 mL of Lysis Buffer 1 (1 M sorbitol, 50 mM Tris-HCl pH 7.2, 10 mM DTT), recovered by centrifugation at 4° and resuspended in 120 mL of Lysis Buffer 2 (1 M sorbitol, 50 mM Tris-HCl pH 7.2, 3 mM DTT, 0.4 mg/mL Zymolyase 20T). The cell suspension was incubated at 30° for 30 min with gentle swirling. Cells were spun at 4000 rpm for 5 min at 4° and washed gently, but thoroughly with 120 mL Lysis Buffer 3 (1 M sorbitol, 10 mM Tris-HCl pH 7.2). The cells were harvested at 4000 rpm for 5 min at 4° (using the same rotor as above), followed by resuspension in 60 mL CBM3 binding buffer (50 mM Tris-HCl pH 6.5, 100 mM NaCl, 1 mM EDTA) (Wan *et al.* 2011) containing cOmplete, Mini, EDTA-free Protease Inhibitor Cocktail (Roche, Basel, Switzerland). The suspension was sonicated (Branson Digital Sonifier 450 using the 102-C converter, 1/2” tapped bio horn, and 1/8” tapered microtip as the probe assembly) on ice for a total of 1.5 min (3 sec on, 10 sec off) at 25% amplitude. Debris was spun out at 4000 rpm for 5 min at 4° and lysate was then aliquoted for binding experiments and stored at –80°.

### Preparation of regenerated amorphous cellulose (RAC)

RAC was prepared by acid treatment as previously described (Wang and Hong 2014). The resulting material is rather gelatinous and can be difficult to work with. After the acid treatment, the material was resuspended once in CBM3 binding buffer with 2M salt, once in CBM3 binding buffer with no salt, and three times in CBM3 binding buffer. The RAC was resuspended and stored in a 1:1 slurry of cellulose and binding buffer.

### Preparation of microgranular cellulose

Cellulose was washed three times in a 20 volume excess of 2 M NaCl CBM3 binding buffer, 2 times in no salt CBM3 binding buffer, and three times in CBM3 binding buffer. Cellulose was resuspended in a 1:1 slurry with CBM3 binding buffer.

### Cellulose pulldowns

For each pulldown reaction, 450 µL of cell lysate (as prepared above) was used to resuspend variable volumes of packed cellulose pellet and allowed to bind at 4° for 20 min with gentle agitation. Cellulose pellets were washed four times in an excess volume (50-fold) of binding buffer before elution in 50 µL of SDS-PAGE Loading Buffer (1× working: 80 mM Tris-HCl pH 6.8, 10% glycerol, 2% SDS, 100 mM DTT, 0.2% bromophenol blue, 100 mM 2-Mercaptoethanol), a binding buffer with varied additives, or a TEV protease elution buffer.

### TEV protease elution

TEV protease was purified using a previously described plasmid encoding a His6-tagged TEV clone and published protocol (Tropea *et al.* 2009). TEV elutions were done in 100 µL of TEV protease buffer (50 mM Tris-HCl pH 8.0, 0.5 mM EDTA, 1 mM DTT) containing varying amounts of TEV protease at 30° for 1 hr. Units of TEV were determined by comparison to AcTEV protease (Life Technologies, Carlsbad, CA). TEV protease was removed by the PureProteome Nickel Magnetic Bead System (EMD Millipore, Billerica, MA) following the manufacturer’s instructions (http://www.emdmillipore.com/US/en/product/PureProteome%E2%84%A2-Nickel-Magnetic-Bead-System,MM_NF-LSKMAGH02?bd=1#documentation).

### Protein visualization and quantification

For western blot analysis, SDS-PAGE loading buffer was added to each sample and heated at 95° for 5 min, spun briefly in a microcentrifuge to remove debris, and subjected to electrophoresis on 4–15% gradient, 15 well SDS-PAGE gels (BioRad, Hercules, CA). The general western blot protocol at (http://www.abcam.com/protocols/general-western-blot-protocol) was used for all western blots. PVDF membrane (EMD Millipore, Billerica, MA) was used for all transfers. Primary antibody incubation was done with a 1:400 dilution of an anti-Myc-repeat antibody (9E10) (Santa Cruz Biotech, Dallas, TX) for 1 hr at 4°, and a secondary antibody incubation was performed with a 1:1000 dilution of ECL anti-mouse IgG, HRP conjugate (GE Healthcare, Little Chalfont, United Kingdom) for 1 hr at room temperature. Pierce ECL Western Blotting Substrate (Thermo Scientific, Waltham, MA) was used according to the manufacturer’s instructions for visualization and the blots were subsequently exposed to x-ray film and developed on a Konica Minolta SRX-101A developer. Protein gels were stained using Coomassie Brilliant Blue R-250 (BioRad, Hercules, CA) according to the manufacturer’s specifications (http://www.bio-rad.com/en-us/product/coomassie-stains). Protein concentration was determined using the DC Protein Assay kit (BioRad, Hercules, CA) microplate protocol (http://www.bio-rad.com/LifeScience/pdf/Bulletin_9005.pdf).

### Data availability

CelTag plasmid is available via Addgene (Plasmid #66562). Full plasmid sequence is available in File S1 and from Addgene. Figure S1 shows the full plasmid map, and Figure S2 shows the annotated CelTag sequence with primer alignments. Primers used in this study are listed in Table S1.

## Results

### Yeast Pgk1 fused with the CelTag binds efficiently to cellulose

**Figure 2:**
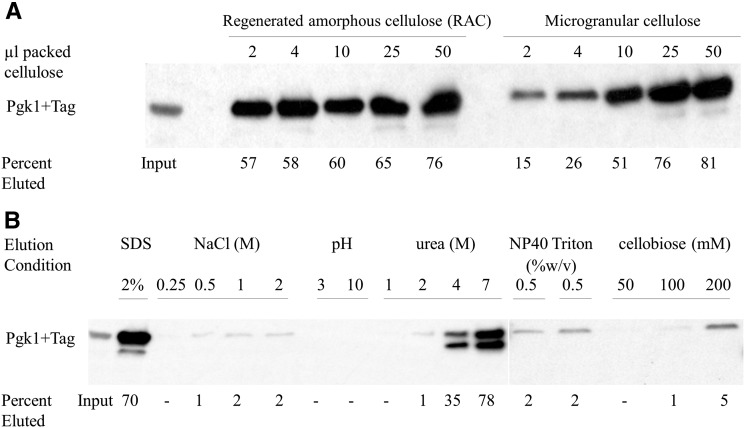
Titration of cellulose and resistance to elution conditions. (A) Titration of the amount and type of cellulose affinity support. Pulldowns were done using 450 µL of lysate and varying amounts of packed microgranular cellulose or regenerated amorphous cellulose (RAC). Binding was done at 4° for 20 min with gentle mixing, after which the cellulose was washed extensively with binding buffer and the bound protein eluted in 50 µL of SDS-PAGE Loading Buffer. Protein was visualized by western blot analysis using a c-Myc (9E10) antibody. As previously described (Hong *et al.* 2007, 2008; Wang and Hong 2014), the regenerated amorphous cellulose binds more CBM3 (family 3 cellulose binding module) per ml of packed resin than the untreated microgranular cellulose. However, higher amounts of microgranular cellulose also efficiently bind the target protein. Since the cost of microgranular cellulose is low and RAC is more difficult to make and work with, subsequent studies were done with only washed, commercial microgranular cellulose. (B) CelTag binding to cellulose is resistant to stringent washing conditions. Removal of nonspecific or loosely bound components before elution could improve specificity or stringency in some applications. CelTag-cellulose binding stability was tested under various conditions. Protein from 450 µL of yeast cell lysate was bound to 50 µL of microgranular cellulose and washed as above with binding buffer. The cellulose was then eluted with an equal volume (50 µL) of binding buffer containing various pH levels, salt concentrations, urea concentrations, nonionic detergents, or the cellobiose dimer unit of cellulose as a possible competitive elution. Only urea eluted levels of protein similar to those obtained with 2% SDS, and even then only at concentrations over 2 M.

To test whether a protein fused with the CelTag peptide sequences on its C-terminal end could efficiently bind to cellulose, we chromosomally tagged the ORF for Pgk1, a soluble cytoplasmic protein, as a proof of concept. The CelTag DNA sequences were chromosomally integrated onto the C-terminus of the *PGK1* gene using homologous recombination (Figure 1A), allowing the purification of tagged protein using cellulose as the affinity matrix, eluting from the matrix with TEV protease or SDS (Figure 1B). Previously, it was shown that commercially available microgranular cellulose powder has suboptimal accessible surface area and binding capacity compared to regenerated amorphous cellulose (RAC) made by phosphoric acid treatment of the microgranular material (Hong *et al.* 2007, 2008; Wang and Hong 2014). Although this treatment causes the cellulose to form more gel-like pellets and is somewhat burdensome for regular use, we compared whether microgranular cellulose or RAC more efficiently pulled down Pgk1-CelTag to a degree that would justify its use. Pgk1-CelTag was pulled down from 450 µL of extract with varying amounts of both regenerated amorphous cellulose and untreated microgranular cellulose. After binding for 20 min at 4° and extensive washing, the cellulose pellets were eluted in 50 µL of SDS-PAGE Loading Buffer and subsequently run on a protein gel (Figure 2A). The results confirm that RAC is more efficient at pulling down the tagged protein, though microgranular cellulose is at least as efficient as RAC at recovering tagged protein (76% from RAC *vs.* 81% from microgranular at the highest concentrations used, Figure 2A). This obviates the need for RAC, since it is more difficult to use, and thus, the subsequent experiments were done using microgranular cellulose. The elutions that gave the most tagged protein from 450 µL of cell extract were the packed pellet of 25 µL or 50 µL microgranular cellulose. Thus, subsequent experiments used approximately 1/20 volume packed cellulose relative to extract volume. Tagged protein was not detected in the unbound fractions (data not shown) suggesting that some protein might be unrecoverable from the cellulose matrix.

### Pgk1-CelTag bound to cellulose is resistant to stringent wash conditions

Next, we sought to determine under what wash conditions the tagged protein would be able to remain bound to the cellulose. Part of the utility of the CelTag could be the high affinity of the CBM3 tag for cellulose under a variety of conditions, allowing stringent washing of the bound protein complexes to remove loosely or nonspecifically bound contaminants. Fifty µL of packed microgranular cellulose was used to pull down tagged protein in 450 µL of lysate. Pellets were eluted in 50 µL of binding buffer containing various additions. CelTag binding seemed to be relatively resistant to high salt elutions. Even in the presence of 2 M NaCl, only about 2% of the tagged protein in the input was eluted, indicating that a majority of the tagged protein remains bound to the cellulose (Figure 2B). The tagged protein bound to cellulose was also resistant to elutions in up to 2 M urea with only about 1% of Pgk1-CelTag eluting, compared to 4M urea where about 35% was eluted (Figure 2B). Moderate acidic and basic conditions (pH 3 or 10) also eluted only small amounts of protein (Figure 2B) and binding was almost completely resistant to concentrations of the nonionic detergents NP-40 and Triton X-100 above their critical micelle concentrations, with only 2% of the tagged protein eluted in each case (Figure 2B). Lastly, we investigated whether cellobiose, the cellulose sugar dimer, is able to competitively elute the protein from the matrix. At concentrations just below saturation, cellobiose was only able to elute 5% of the total protein off the cellulose over the time course used (Figure 2B). These data suggest that various stringent washing conditions might be used prior to elution.

### TEV protease efficiently and specifically elutes Pgk1-CelTag from cellulose

**Figure 3:**
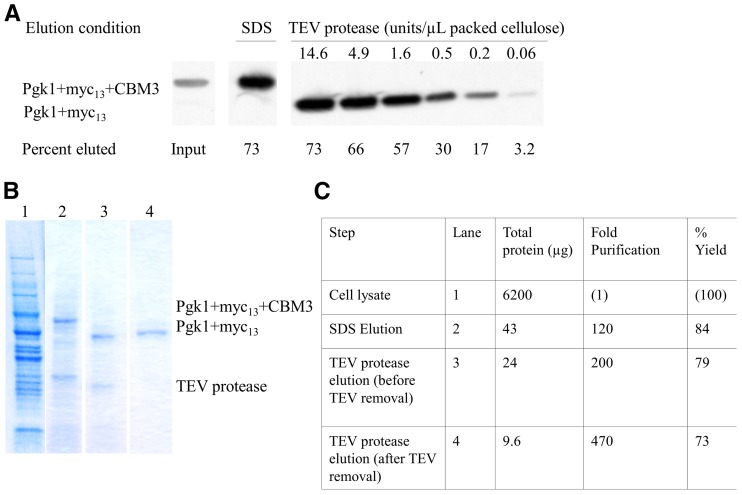
TEV protease elution from cellulose is specific and provides highly purified target protein. (A) Titration of TEV (Tobacco Etch Virus) protease purified in the lab as previously described (Tropea *et al.* 2009). In each lane, protein from 450 µL yeast cell extract was bound to 50 µL microgranular cellulose and eluted with TEV protease in 100 µL of elution buffer (50 mM Tris-HCl pH 8.0, 0.5 mM EDTA, 1 mM DTT) at 30° for 1 hr. Eluates (8 µL) in 2% SDS (sodium dodecyl sulfate) or TEV protease were analyzed by western blot as above. For comparison, 8 µL input cell extract is shown. (B) Denaturing protein gel analysis of the stages of purification, stained with Coomassie Brilliant Blue. Lane 1: 8 µL cell lysate out of 450 µL input. Lane 2: 8 µL (out of 50 µL) SDS elution of cellulose pellet. Lane 3: 8 µL (out of 50 µL) TEV protease elution before nickel bead removal of TEV protease. Lane 4: 8 µL (out of 50 µL) TEV protease elution after nickel bead treatment. (C) Table showing the total protein at each stage of purification, given as the average of three bindings and elutions. Yield of Cel-Tagged protein was determined by average of triplicate samples by western blot analysis.

We next determined the approximate amount of TEV protease needed for optimal release of tagged protein from the cellulose. At the highest concentration of TEV protease, 73% of the tagged protein relative to input was eluted from the cellulose (indicated by a smaller band representing the Pgk1 with the cellulose binding module removed) (Figure 3A, lanes 1 and 3). The TEV elution was also very specific and eliminated most of the residual nonspecific binding contamination seen if the pellet is eluted in SDS (Figure 3B, lanes 2 and 3). To remove the recombinant His6-tagged TEV protease from the elution, we passed the eluted sample through nickel-coated beads (Figure 3B, lane 3 and 4). The total yield and fold-purification using the above method to purify a CelTagged protein is summarized in Figure 3C. In our hands, the CelTag purification scheme is able to provide ∼470-fold purification of the protein of interest, more importantly recovering about 73% of the total starting protein.

## Discussion

We have shown as a proof of principle that the CelTag affinity tag is able to efficiently recover the majority of an endogenous, tagged protein from crude cellular extracts with nearly complete purity. In contrast to previous uses of the cellulose tags, it appears that using commercially available microgranular cellulose is as effective as using the more labor-intensive RAC at binding the CBM3 domain as an affinity tag. This is useful since microgranular cellulose is much easier to manipulate and does not require acid treatment. The recombinant DNA construct encoding the CelTag has been designed to also contain a 13x c-Myc epitope tag, so that the fragment to be used for tagging a chromosomal ORF can be lifted by PCR to either contain or delete the c-Myc portion as an additional isolation or detection tool.

Another useful aspect of the CelTag is that relatively stringent washing conditions are possible once the tagged proteins are bound to the cellulose support. High salt, nonionic detergents and urea were all tested and showed that reasonably severe, nondenaturing conditions can be used to minimize nonspecific or loosely associated macromolecules from the isolated target. This is in contrast to previous purifications using the commercially available TAP tag. Typical recovery using mild conditions results in an average loss of 70–80% in yeast (Li 2011), whereas we observed 1–5% loss of total tagged protein even under much harsher conditions. We note that stringent washing conditions might also remove specific partners from a complex and conditions would need to be optimized for individual applications. Cellobiose elutions were attempted to see whether the tag could be competed off the cellulose without the use of TEV protease, but with soluble concentrations of cellobiose the tag is unable to be competed off within the tested time frame, likely due to the higher affinity and specificity that the CelTag has for the cellulose polymer. Therefore, TEV protease was optimized for efficient elution of CelTagged protein off cellulose.

For this proof of concept for using cellulose as an affinity resin, we have used a relatively abundant, soluble enzyme that was not necessarily expected to have stably associated partners in a physiological complex. For rare proteins and those found in extended complexes, it might be challenging to distinguish between background contaminants and *bona fide* binding partners. It is possible that *in vivo* crosslinking might be used to support associations that occur in the cells, as is currently used for a wide variety of complexes (*e.g.*, chromatin immunoprecipitation) with other affinity tags. It is anticipated that the stability of the CelTag-cellulose binding to stringent washing might be useful in these contexts.

## Supplementary Material

Supporting Information
