# Supplementary material for: A Novel Recombinant DNA System for High Efficiency Affinity Purification of Proteins in Saccharomyces cerevisiae
Source: G3 (Bethesda). 2015 Dec 29;6(3):573–8. doi: 10.1534/g3.115.025106 (PMC4777120; doi:10.1534/g3.115.025106)
Supplement: Supporting Information [file supp_6_3_573__index.html]

A Novel Recombinant DNA System for High Efficiency Affinity Purification of Proteins in Saccharomyces cerevisiae — Supporting Information 

# A Novel Recombinant DNA System for High Efficiency Affinity Purification of Proteins in *Saccharomyces cerevisiae*

## Supporting Information for Carrick *et al.*, 2016

**Files in this Data Supplement:**

- Figure S1 - Plasmid map. (.pdf, 256 KB)
- Figure S2 - CelTag fragment sequence with primer alignment. (.pdf, 561 KB)
- Table S1 - Primer sequences. (.pdf, 64 KB)
- File S1 - Complete plasmid sequence. (.pdf, 14 KB)
