# Supplementary figures and images for: A Novel Recombinant DNA System for High Efficiency Affinity Purification of Proteins in Saccharomyces cerevisiae
Source: G3 (Bethesda). 2015 Dec 29;6(3):573–8. doi: 10.1534/g3.115.025106 (PMC4777120; doi:10.1534/g3.115.025106)

**Figure S1**  
**Plasmid Map**

Created with SnapGene®

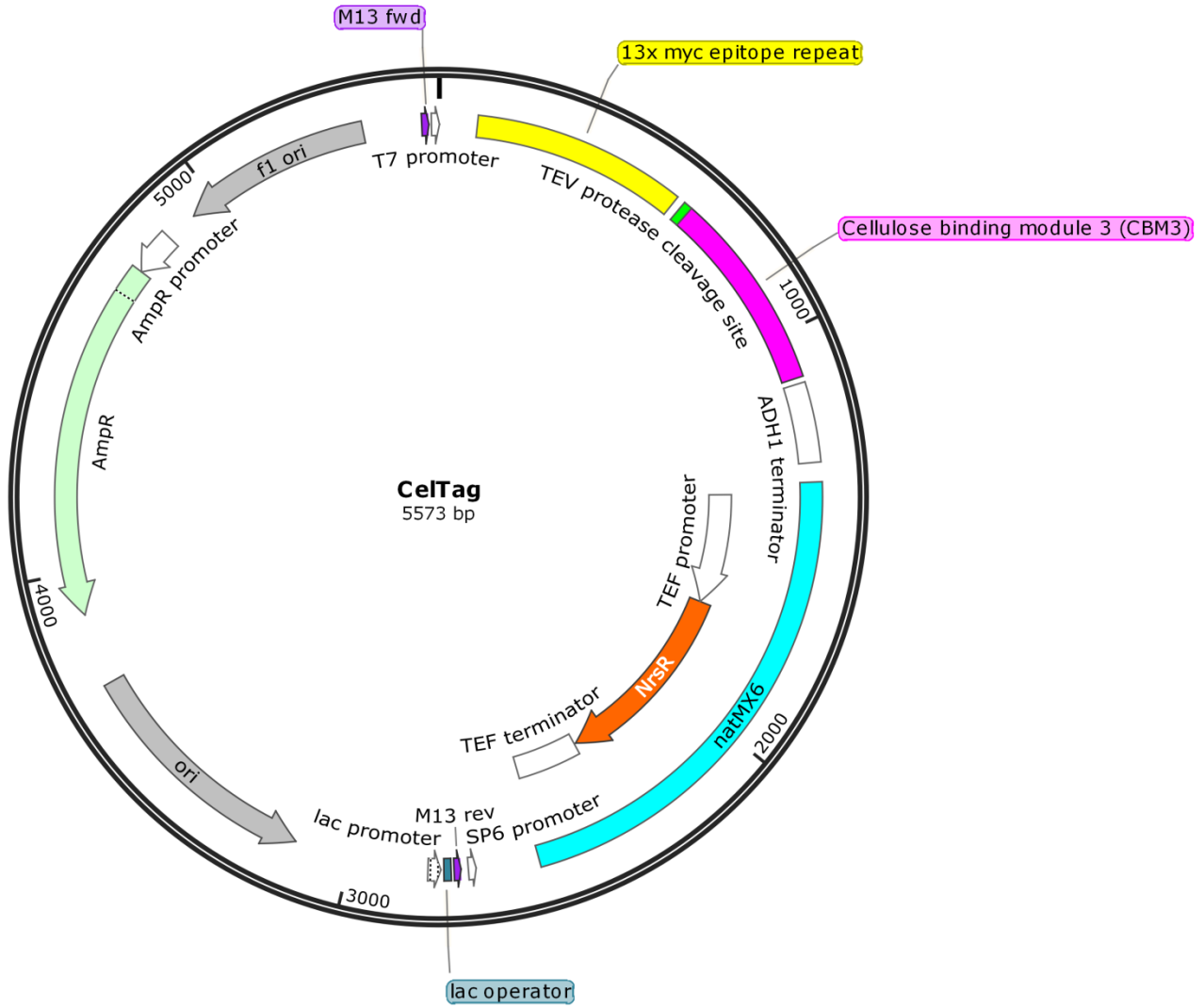

Supplement: Supporting Information [file supp_g3.115.025106_FigureS1.pdf]
