# Supplementary material for: A Novel Recombinant DNA System for High Efficiency Affinity Purification of Proteins in Saccharomyces cerevisiae
Source: G3 (Bethesda). 2015 Dec 29;6(3):573–8. doi: 10.1534/g3.115.025106 (PMC4777120; doi:10.1534/g3.115.025106)
Supplement: Supporting Information [file supp_g3.115.025106_FigureS2.pdf]

Figure S2

CelTag Fragment Sequence with Primer Alignment

AATCGATACATATGCCCGGGTTAATTAACGGTGAACAAAAGCTAATCTCCGAGGAAGACTTGAACGGTGAACAAAAATTAATCTCAGAAGAAGACTTGAACGGACTC
GACGGTGAACAAAAGTTGATTTCTGAAGAAGATTTGAACGGTGAACAAAAGCTAATCTCCGAGGAAGACTTGAACGGTGAACAAAAATTAATCTCAGAAGAAGAC
TTGAACGGACTCGACGGTGAACAAAAGTTGATTTCTGAAGAAGATTTGAACGGTGAACAAAAGCTAATCTCCGAGGAAGACTTGAACGGTGAACAAAAATTAATCT
CAGAAGAAGACTTGAACGGACTCGACGGTGAACAAAAGTTGATTTCTGAAGAAGATTTGAACGGTGAACAAAAGCTAATCTCCGAGGAAGACTTGAACGGTGAAC
AAAAATTAATCTCAGAAGAAGACTTGAACGGACTCGACGGTGAACAAAAGTTGATTTCTGAAGAAGATTTGAACGGTGAACAAAAGCTAATCTCCGAGGAAGACTT
GAACGGTGAACAAAAATTAATCAATCACGAGAACCTCTACTTCCAAAGCGTATCAGGCAATTTGAAGGTTGAATTCTACAACAGCAATCCTTCAGATACTACTAACTC
AATCAATCCTCAGTTCAAGGTTACTAATACCGGAAGCAGTGCAATTGATTTGTCCAAACTCACATTGAGATATTATTATACAGTAGACGGACAGAAAGATCAGACCTTC
TGGTGTGACCATGCTGCAATAATCGGCAGTAACGGCAGCTACAACGGAATTACTTCAAATGTAAAAGGAACATTTGTAAAAATGAGTTCCTCAACAAATAACGCAGA
CACCTACCTTGAAATAAGCTTTACAGGCGGAACTCTTGAACCGGGTGCACATGTTCAGATACAAGGTAGATTTGCAAAGAATGACTGGAGTAACTATACACAGTCAA
ATGACTACTCATTCAAGTCTGCTTCACAGTTTGTTGAATGGGATCAGGTAACAGCATACTTGAACGGTGTTCTTGTATGGGGTAAAGAATAGACTTCTAAATAAGCGA
ATTTCTTATGATTTATGATTTTATTATTAAATAAGTTATAAAAAAAATAAGTGTATACAAATTTTAAAGTGACTCTTAGGTTTTAAAACGAAAATTCTTATTCTTGAGTAA
CTCTTTCCTGTAGGTCAGGTTGCTTTCTCAGGTATAGTATGAGGTCGCTCTTATTGACCACACCTCTACCGGCAGATCCGCTAGGGATAACAGGGTAATATAGATCTGTT
TAGCTTGCCTCGTCCCCGCCGGGTCACCCGGCCAGCGACATGGAGGCCCAGAATACCCTCCTTGACAGTCTTGACGTGCGCAGCTCAGGGGCATGATGTGACTGTCG
CCCGTACATTTAGCCCATACATCCCCATGTATAATCATTTGCATCCATACATTTTGATGGCCGCACGGCGCGAAGCAAAAATTACGGCTCCTCGCTGCAGACCTGCGAG
CAGGGAAACGCTCCCCTCACAGACGCGTTGAATTGTCCCCACGCCGCGCCCCTGTAGAGAAATATAAAAGGTTAGGATTTGCCACTGAGGTTCTTCTTTCATATACTT
CCTTTTTAAATCTTGCTAGGATACAGTTCTCACATCACATCCGAACATAAACAACCATGGGTACCACTCTTGACGACACGGCTTACCGGTACCGCACCAGTGTCCCGG
GGGACGCCGAGGCCATCGAGGCACTGGATGGGTCCTTCACCACCGACACCGTCTTACTGGATGGGTCCTTCACCACCGACACCGTCTTCCGCGTCACCGCCACCGG
GGACGGCTTCACCCTGCGGGAGGTGCCGGTGGACCCGCCCCTGACCAAGGTGTTCCCCGACGACGAATCGGACGACGAATCGGACGACGGGGAGGACGGCGACCC
GGACTCTCGGACGTTCGTCGCGTACGGGGACGACGGCGACCTGGCGGGCTTCGTGGTCGTCTCGTACTCCGGCTGGAACCGCCGGCTGACCGTCGAGGACATCGAG
GTCGCCCCGGAGCACCGGGGGCACGGGGTCGGGCGCGCGTTGATGGGGCTCGCGACGGAGTTCGCCCGCGAGCGGGGTGCCGGGCACCTCTGGCTGGAGGTCACC
AACGTCAACGCACCGGCGATCCACGCGTACCGGCGGATGGGGTTCACCCTCTGCGGCCTGGACACCGCCCTGTACGACGGCACCGCCTCGGACGGCGAGCAGGCG
CTCTACATGAGCATGCCCTGCCCCTAATCAGTACTGACAATAAAAAGATTCTTGTTTTCAAGAACTTGTCATTTGTATAGTTTTTTTATATTGTAGTTGTTCTATTTTAAT
CAAATGTTAGCGTGATTTATATTTTTTTTCGCCTCGACATCATCTGCCCAGATGCGAAGTTAAGTGCGCAGAAAGTAATATCATGCGTCAATCGTATGTGAATGCTGGTC
GCTATACTGCTGTCGATTCGATACTAACGCCGCCATCCAGTTTAAACGAGCTCGAATTCATCGATACCGTCGACCTCGAGCGTACGTA

Primer	Sequence (5'-3')
Forward including 13x myc epitope repeat	5'-50 base pair homology to end of protein orf without stop codon CCCGGGTTAATTAACGGTGAA-3'
Forward excluding 13x myc epitope repeat	5'-50 base pair homology to end of protein orf without stop codon GAGAACCTCTACTTCCAAAGC-3'
Reverse	5'-50bp homology 25 base pairs downstream of target orf (reverse complement) ATGAATTCGAGCTCGTTTAAA-3'

Figure S2: Sequence of the CelTag fragment showing primer design strategy. Yellow is the 13x myc repeat, green indicates the TEV protease site, purple is the CBM3domain, and blue-green is the selection marker, *nat,1* including its promoter and terminator sequences.
