# Supplementary material for: A Novel Recombinant DNA System for High Efficiency Affinity Purification of Proteins in Saccharomyces cerevisiae
Source: G3 (Bethesda). 2015 Dec 29;6(3):573–8. doi: 10.1534/g3.115.025106 (PMC4777120; doi:10.1534/g3.115.025106)
Supplement: Supporting Information [file supp_g3.115.025106_TableS1.pdf]

Table S1

Primer Sequences

**Primer**	**Sequence 5’-3’**
CelTag Fragment with PGK1 homologous ends (end of ORF)	**5’-**TTATTGGAAGGTAAGGAATTGCCAGGTGTTGCTTTCTTATCCGAAAAGAAACCCGGGTTAATTAACGGTGAA-**3’**
CelTag Fragment with PGK1 homologous ends (downstream of ORF)	**5’**TATTATTTTAGCGTAAAGGATGGGGAAAGAGAAAAGAAAAAAATTGATCTAATGAATTCGAGCTCGTTTAAA **-3’**
