# Supplementary material for: A Novel Recombinant DNA System for High Efficiency Affinity Purification of Proteins in Saccharomyces cerevisiae
Source: G3 (Bethesda). 2015 Dec 29;6(3):573–8. doi: 10.1534/g3.115.025106 (PMC4777120; doi:10.1534/g3.115.025106)
Supplement: Supporting Information [file supp_g3.115.025106_FileS1.pdf]

File S1

Complete Plasmid Sequence

GGGCGAATTGGGCCCGACGTCGCATGCTCCCGGCCGCCATGGCGGCCGCGGGAATT
CGATAATCGATACATATGCCCGGGTTAATTAACGGTGAACAAAAGCTAATCTCCGA
GGAAGACTTGAACGGTGAACAAAAAATTAATCTCAGAAGAAGACTTGAACGGACTC
GACGGTGAACAAAAGTTGATTTCTGAAGAAGATTTGAACGGTGAACAAAAGCTAAT
CTCCGAGGAAGACTTGAACGGTGAACAAAAATTAATCTCAGAAGAAGACTTGAACG
GACTCGACGGTGAACAAAAGTTGATTTCTGAAGAAGATTTGAACGGTGAACAAAAG
CTAATCTCCGAGGAAGACTTGAACGGTGAACAAAAATTAATCTCAGAAGAAGACTT
GAACGGACTCGACGGTGAACAAAAGTTGATTTCTGAAGAAGATTTGAACGGTGAAC
AAAAGCTAATCTCCGAGGAAGACTTGAACGGTGAACAAAAATTAATCTCAGAAGAA
GACTTGAACGGACTCGACGGTGAACAAAAGTTGATTTCTGAAGAAGATTTGAACGG
TGAACAAAAGCTAATCTCCGAGGAAGACTTGAACGGTGAACAAAAATTAATCAATC
ACGAGAACCTCTACTTCCAAAGCGTATCAGGCAATTTGAAGGTTGAATTCTACAACA
GCAATCCTTCAGATACTACTAACTCAATCAATCCTCAGTTCAAGGTTACTAATACCG
GAAGCAGTGCAATTGATTTGTCCAAACTCACATTGAGATATTATTATACAGTAGACG
GACAGAAAGATCAGACCTTCTGGTGTGACCATGCTGCAATAATCGGCAGTAACGGC
AGCTACAACGGAATTACTTCAAATGTAAAAGGAACATTTGTAAAAATGAGTTCCTCA
ACAAATAACGCAGACACCTACCTTGAAATAAGCTTTACAGGCGGAACTCTTGAACC
GGGTGCACATGTTCAGATACAAGGTAGATTTGCAAAGAATGACTGGAGTAACTATA
CACAGTCAAATGACTACTCATTCAAGTCTGCTTCACAGTTTGTTGAATGGGATCAGG
TAACAGCATACTTGAACGGTGTTCTTGTATGGGGTAAAGAATAGACTTCTAAATAAG
CGAATTTCTTATGATTTATGATTTTTATTATTAAATAAGTTATAAAAAAAATAAGTGT
ATACAAATTTTAAAGTGACTCTTAGGTTTTAAAACGAAAATTCTTATTCTTGAGTAA
CTCTTTCCTGTAGGTCAGGTTGCTTTCTCAGGTATAGTATGAGGTCGCTCTTATTGAC
CACACCTCTACCGGCAGATCCGCTAGGGATAACAGGGTAATATAGATCTGTTTAGCT
TGCCTCGTCCCCGCCGGGTCACCCGGCCAGCGACATGGAGGCCCAGAATACCCTCCT
TGACAGTCTTGACGTGCGCAGCTCAGGGGCATGATGTGACTGTCGCCCGTACATTTA
GCCCATACATCCCCATGTATAATCATTTGCATCCATACATTTTGATGGCCGCACGGC
GCGAAGCAAAAATTACGGCTCCTCGCTGCAGACCTGCGAGCAGGGAAACGCTCCCC
TCACAGACGCGTTGAATTGTCCCCACGCCGCGCCCCTGTAGAGAAATATAAAAGGTT
AGGATTTGCCACTGAGGTTCTTCTTTCATATACTTCCTTTTTAAATCTTGCTAGGATA
CAGTTCTCACATCACATCCGAACATAAACAACCATGGGTACCACTCTTGACGACACG
GCTTACCGGTACCGCACCAGTGTCCCGGGGGACGCCGAGGCCATCGAGGCACTGGA
TGGGTCCTTCACCACCGACACCGTCTTACTGGATGGGTCCTTCACCACCGACACCGT
CTTCCGCGTCACCGCCACCGGGGACGGCTTCACCCTGCGGGAGGTGCCGGTGGACC
CGCCCCTGACCAAGGTGTTCCCCGACGACGAATCGGACGACGAATCGGACGACGGG
GAGGACGGCGACCCGGACTCTCGGACGTTCGTCGCGTACGGGGACGACGGCGACCT
GGCGGGCTTCGTGGTCGTCTCGTACTCCGGCTGGAACCGCCGGCTGACCGTCGAGGA
CATCGAGGTCGCCCCGGAGCACCGGGGGCACGGGGTCGGGCGCGCGTTGATGGGGC
TCGCGACGGAGTTCGCCCGCGAGCGGGGTGCCGGGCACCTCTGGCTGGAGGTCACC
AACGTCAACGCACCGGCGATCCACGCGTACCGGCGGATGGGGTTCACCCTCTGCGG
CCTGGACACCGCCCTGTACGACGGCACCGCCTCGGACGGCGAGCAGGCGCTCTACA
TGAGCATGCCCTGCCCCTAATCAGTACTGACAATAAAAAGATTCTTGTTTTCAAGAA
CTTGTCATTTGTATAGTTTTTTTATATTGTAGTTGTTCTATTTTAATCAAATGTTAGCG

TGATTTATATTTTTTTTCGCCTCGACATCATCTGCCCAGATGCGAAGTTAAGTGCGCA
GAAAGTAATATCATGCGTCAATCGTATGTGAATGCTGGTCGCTATACTGCTGTCGAT
TCGATACTAACGCCGCCATCCAGTTTAAACGAGCTCGAATTCATCGATACCGTCGAC
CTCGAGCGTACGTAATCACTAGTGAATTCGCGGCCGCCTGCAGGTCGACCATATGGG
AGAGCTCCCAACGCGTTGGATGCATAGCTTGAGTATTCTATAGTGTCACCTAAATAG
CTTGGCGTAATCATGGTCATAGCTGTTTCCTGTGTGAAATTGTTATCCGCTCACAATT
CCACACAACATACGAGCCGGAAGCATAAAGTGTAAAGCCTGGGGTGCCTAATGAGT
GAGCTAACTCACATTAATTGCGTTGCGCTCACTGCCCGCTTTCCAGTCGGGAAACCT
GTCGTGCCAGCTGCATTAATGAATCGGCCAACGCGCGGGGAGAGGCGGTTTGCGTA
TTGGGCGCTCTTCCGCTTCCTCGCTCACTGACTCGCTGCGCTCGGTCGTTCGGCTGCG
GCGAGCGGTATCAGCTCACTCAAAGGCGGTAATACGGTTATCCACAGAATCAGGGG
ATAACGCAGGAAAGAACATGTGAGCAAAAGGCCAGCAAAAGGCCAGGAACCGTAA
AAAGGCCGCGTTGCTGGCGTTTTTCCATAGGCTCCGCCCCCCTGACGAGCATCACAA
AAATCGACGCTCAAGTCAGAGGTGGCGAAACCCGACAGGACTATAAAGATACCAGG
CGTTTCCCCCTGGAAGCTCCCTCGTGCGCTCTCCTGTTCCGACCCTGCCGCTTACCGG
ATACCTGTCCGCCTTTCTCCCTTCGGGAAGCGTGGCGCTTTCTCATAGCTCACGCTGT
AGGTATCTCAGTTCGGTGTAGGTCGTTCGCTCCAAGCTGGGCTGTGTGCACGAACCC
CCCGTTCAGCCCGACCGCTGCGCCTTATCCGGTAACTATCGTCTTGAGTCCAACCCG
GTAAGACACGACTTATCGCCACTGGCAGCAGCCACTGGTAACAGGATTAGCAGAGC
GAGGTATGTAGGCGGTGCTACAGAGTTCTTGAAGTGGTGGCCTAACTACGGCTACAC
TAGAAGAACAGTATTTGGTATCTGCGCTCTGCTGAAGCCAGTTACCTTCGGAAAAAG
AGTTGGTAGCTCTTGATCCGGCAAACAAACCACCGCTGGTAGCGGTGGTTTTTTTGT
TTGCAAGCAGCAGATTACGCGCAGAAAAAAAGGATCTCAAGAAGATCCTTTGATCT
TTTCTACGGGGTCTGACGCTCAGTGGAACGAAAACTCACGTTAAGGGATTTTGGTCA
TGAGATTATCAAAAAGGATCTTCACCTAGATCCTTTTAAATTAAAAATGAAGTTTTA
AATCAATCTAAAGTATATATGAGTAAACTTGGTCTGACAGTTACCAATGCTTAATCA
GTGAGGCACCTATCTCAGCGATCTGTCTATTTCGTTCATCCATAGTTGCCTGACTCCC
CGTCGTGTAGATAACTACGATACGGGAGGGCTTACCATCTGGCCCCAGTGCTGCAAT
GATACCGCGAGACCCACGCTCACCGGCTCCAGATTTATCAGCAATAAACCAGCCAG
CCGGAAGGGCCGAGCGCAGAAGTGGTCCTGCAACTTTATCCGCCTCCATCCAGTCTA
TTAATTGTTGCCGGGAAGCTAGAGTAAGTAGTTCGCCAGTTAATAGTTTGCGCAACG
TTGTTGCCATTGCTACAGGCATCGTGGTGTCACGCTCGTCGTTTGGTATGGCTTCATT
CAGCTCCGGTTCCCAACGATCAAGGCGAGTTACATGATCCCCCATGTTGTGCAAAAA
AGCGGTTAGCTCCTTCGGTCCTCCGATCGTTGTCAGAAGTAAGTTGGCCGCAGTGTT
ATCACTCATGGTTATGGCAGCACTGCATAATTCTCTTACTGTCATGCCATCCGTAAG
ATGCTTTTCTGTGACTGGTGAGTACTCAACCAAGTCATTCTGAGAATAGTGTATGCG
GCGACCGAGTTGCTCTTGCCCGGCGTCAATACGGGATAATACCGCGCCACATAGCA
GAACTTTAAAAGTGCTCATCATTGGAAAACGTTCTTCGGGGCGAAAACTCTCAAGGA
TCTTACCGCTGTTGAGATCCAGTTCGATGTAACCCACTCGTGCACCCAACTGATCTTC
AGCATCTTTTACTTTCACCAGCGTTTCTGGGTGAGCAAAAACAGGAAGGCAAAATGC
CGCAAAAAAGGGAATAAGGGCGACACGGAAATGTTGAATACTCATACTCTTCCTTTT
TCAATATTATTGAAGCATTTATCAGGGTTATTGTCTCATGAGCGGATACATATTTGA
ATGTATTTAGAAAAATAAACAAATAGGGGTTCCGCGCACATTTCCCCGAAAAGTGC
CACCTGATGCGGTGTGAAATACCGCACAGATGCGTAAGGAGAAAATACCGCATCAG
GAAATTGTAAGCGTTAATATTTTGTTAAAATTCGCGTTAAATTTTTGTTAAATCAGCT
CATTTTTTAACCAATAGGCCGAAATCGGCAAAATCCCTTATAAATCAAAAGAATAGA

CCGAGATAGGGTTGAGTGTTGTTCCAGTTTGGAACAAGAGTCCACTATTAAAGAACG
TGGACTCCAACGTCAAAGGGCGAAAAACCGTCTATCAGGGCGATGGCCCACTACGT
GAACCATCACCCTAATCAAGTTTTTTGGGGTCGAGGTGCCGTAAAGCACTAAATCGG
AACCCTAAAGGGAGCCCCCGATTTAGAGCTTGACGGGGAAAGCCGGCGAACGTGGC
GAGAAAGGAAGGGAAGAAAGCGAAAGGAGCGGGCGCTAGGGCGCTGGCAAGTGTA
GCGGTCACGCTGCGCGTAACCACCACACCCGCCGCGCTTAATGCGCCGCTACAGGG
CGCGTCCATTCGCCATTCAGGCTGCGCAACTGTTGGGAAGGGCGATCGGTGCGGGC
CTCTTCGCTATTACGCCAGCTGGCGAAAGGGGGATGTGCTGCAAGGCGATTAAGTTG
GGTAACGCCAGGGTTTTCCCAGTCACGACGTTGTAAAACGACGGCCAGTGAATTGTA
ATACGACTCACTATA
